# P-1376. Ethionamide and Ofloxacin-based Antitubercular Therapy for the Treatment of Drug-sensitive Central Nervous System Tuberculosis in Children

**DOI:** 10.1093/ofid/ofaf695.1563

**Published:** 2026-01-11

**Authors:** Dhruv Gandhi, Viren Amesur, Aditi Gupta, Minnie Bodhanwala, Ira Shah

**Affiliations:** Bai Jerbai Wadia Hospital for Children, Mumbai, India, West Monroe, LA; Bai Jerbai Wadia Hospital for Children, Mumbai, India, West Monroe, LA; Bai Jerbai Wadia Hospital for Children, Mumbai, India, West Monroe, LA; Wadia Group of Hospitals, Mumbai, India, Mumbai, Maharashtra, India; Bai Jerbai Wadia Hospital for Children, Mumbai, India, West Monroe, LA

## Abstract

**Background:**

Conventional first-line regimens for pediatric central nervous system tuberculosis (CNS-TB) consisting of isoniazid (H), rifampicin (R), pyrazinamide (Z), and ethambutol (E) have been associated with poor patient outcomes. In view of their bactericidal effect and high penetration rates into the CNS, ethionamide and ofloxacin may improve outcomes in such patients. The aim of this study is to determine treatment outcomes of drug-sensitive pediatric CNS-TB with ethionamide and/or ofloxacin containing first-line regimens along with HRZE.

**Table 1:**
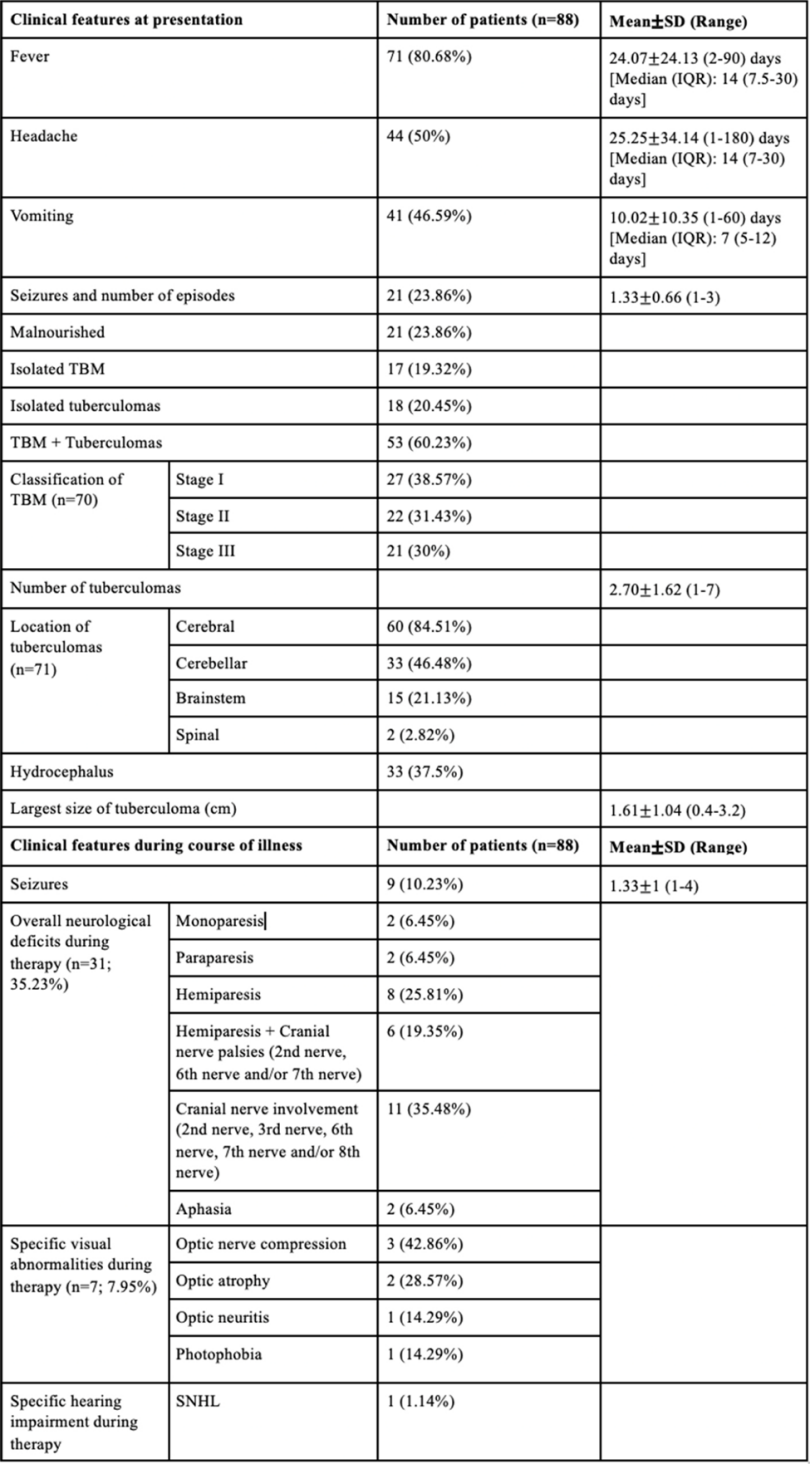
Clinical features of the patients at the time of presentation and during the course of illness Note: SD- Standard deviation, IQR- interquartile range, TBM- tuberculous meningitis, SNHL- Sensorineural hearing loss.

**Table 2:**
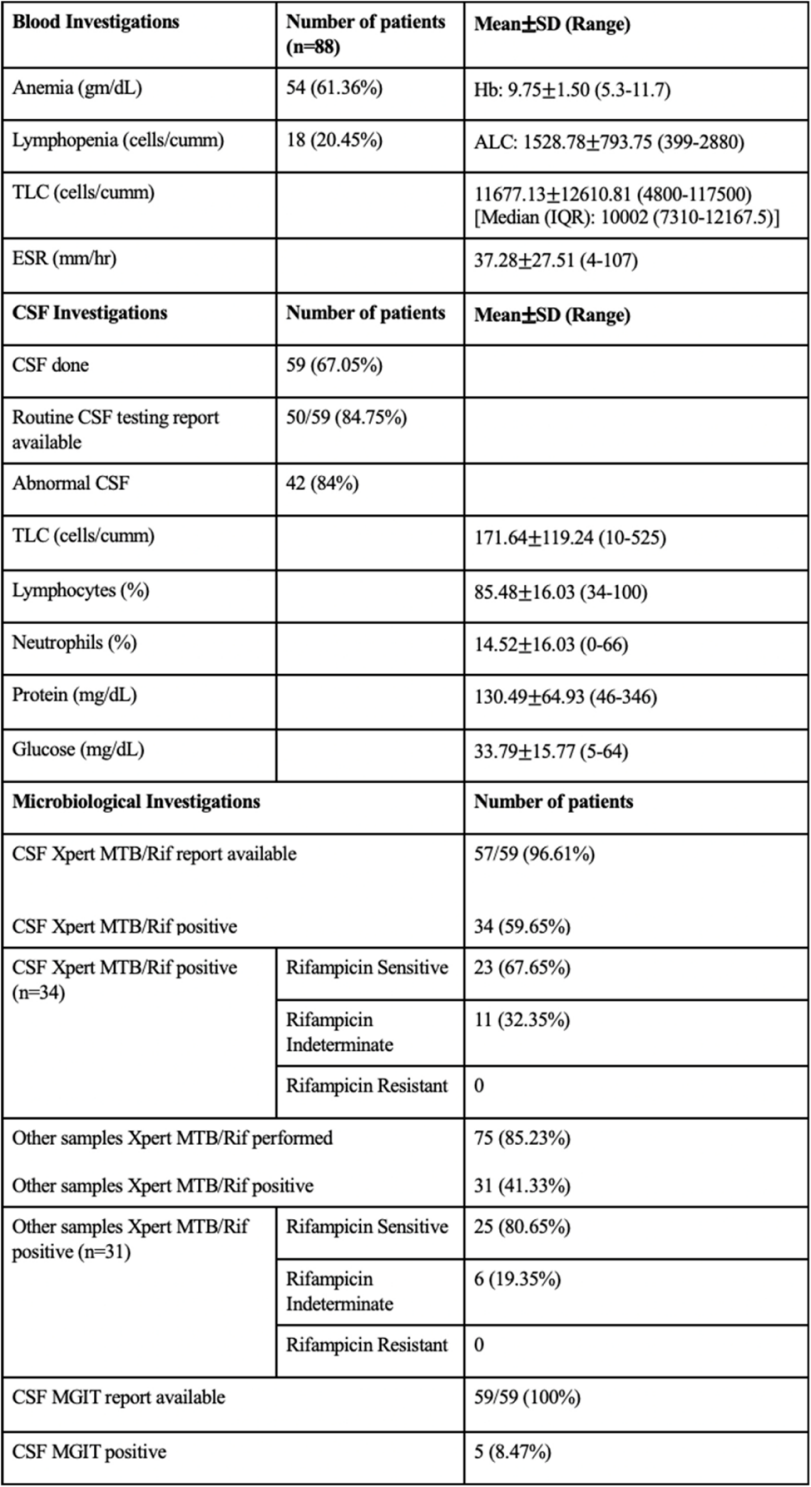
Blood, CSF and microbiological investigations of the patients Note: SD- Standard deviation, Hb: Hemoglobin, ALC: Absolute lymphocyte count, TLC- Total leukocyte count, IQR- Interquartile range, ESR- Erythrocyte sedimentation rate, CSF- Cerebrospinal fluid, MTBC- Mycobacterium tuberculosis complex, MGIT- Mycobacterial Growth Indicator Tube.

**Methods:**

A retrospective study was conducted from March 2020 to August 2024 and included 88 children less than 18 years of age, diagnosed with drug-sensitive CNS-TB, treated with first-line regimens containing ethionamide and/or ofloxacin, and who were followed up in the outpatient clinic. Data on clinicoradiological parameters, adverse effects, and treatment outcomes was analyzed.

**Table 3:**
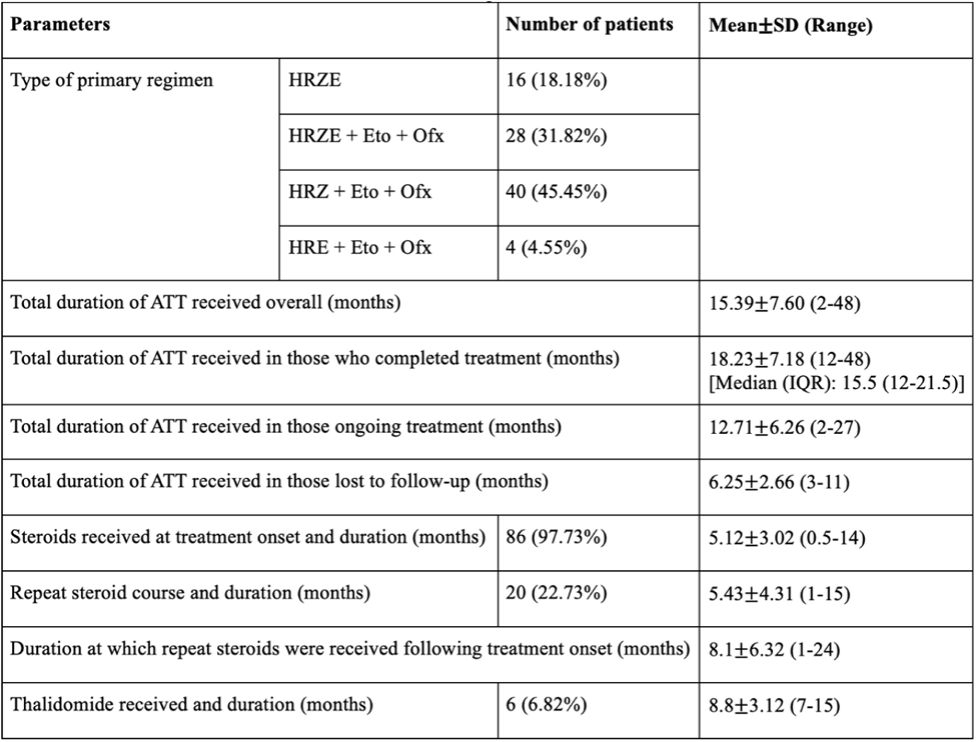
Treatment details of the patients Note: SD- Standard deviation, H- Isoniazid, R-Rifampicin, Z-Pyrazinamide, E-Ethambutol, Eto- Ethionamide, Ofx- Ofloxacin, ATT- Antitubercular therapy, DILI- Drug induced liver injury, IQR- Interquartile range.

**Table 4:**
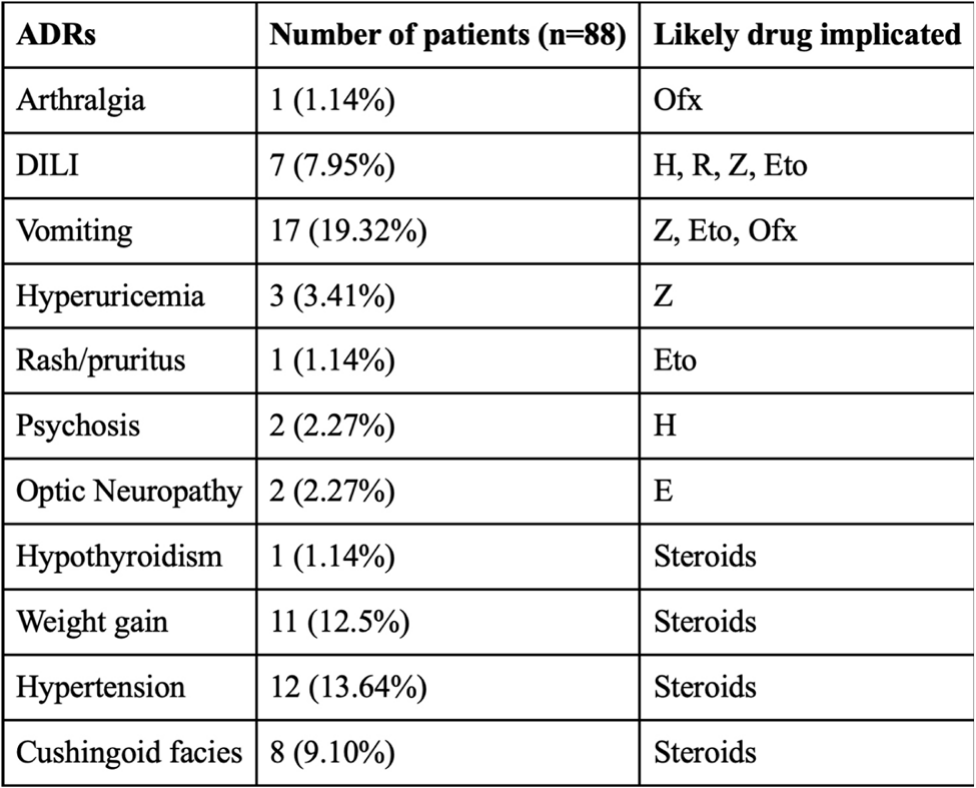
ADRs of antitubercular therapy and steroids Note: ADRs- adverse drug reactions, Ofx- Ofloxacin, DILI- drug induced liver injury, H- Isoniazid, R- Rifampicin, Z-Pyrazinamide, Eto- Ethionamide, CYS- cycloserine, E- Ethambutol.

**Results:**

The mean age at presentation was 6.94±4.48 years. Seventeen (19.3%) patients had isolated TB meningitis, 18 (20.45%) had isolated tuberculomas, and 53 (60.23%) had both. New tuberculomas developed in 33 (37.5%) patients at 6.06±4.89 months of treatment. Treatment failure was seen in 2 (2.28%) patients, who needed to be shifted to second-line antitubercular therapy (ATT). The mean duration of ATT received was 18.23±7.18 months. Fifty-two (59.09%) patients completed treatment, of which 27 (51.92%) completely recovered with normal imaging, 23 (46.15%) completely recovered with persistent tuberculomas and 2 (1.92%) recovered with residual motor deficits. Twenty-eight (31.82%) patients were ongoing treatment and 8 (9.09%) patients were lost to follow-up. Neurological deficits were seen in 7 (7.95%) patients at treatment completion, with 1 (1.14%) patient having bilateral sensorineural hearing loss, 2 (2.27%) patients having bilateral optic atrophy, and 4 (4.55%) patients with motor and cranial nerve palsies. No patient died on follow-up.

**Conclusion:**

Ethionamide and/or ofloxacin containing first-line regimens can achieve favorable neurological outcomes at the end of treatment and may be considered in the management of pediatric CNS-TB. However, treatment duration may be prolonged.

**Disclosures:**

All Authors: No reported disclosures

